# Long-term mental and physical quality of life outcomes following ileal pouch anal anastomosis surgery

**DOI:** 10.1308/rcsann.2023.0075

**Published:** 2024-02-16

**Authors:** M Abdalkoddus, J Franklyn, S Balasubramanya, F Parker, Z Zhao, W Douie, S Smolarek

**Affiliations:** University Hospitals Plymouth NHS Trust, UK

**Keywords:** Inflammatory bowel disease (IBD), Familial adenomatous polyposis (FAP), Ileal pouch anal anastomosis (IPAA), Quality of life

## Abstract

**Introduction:**

This study presents the authors’ experience over 14 years of performing restorative procto-colectomy with ileal pouch anal anastomosis (IPAA). The aim was to study the long-term quality of life outcomes and analyse the predictors of pouch function as well as physical and mental wellbeing.

**Methods:**

This is a single-centre retrospective study conducted in a specialised colorectal surgery unit in the UK. The study included patients who underwent two- or three-staged panproctocolectomy with defunctioning ileostomy for ulcerative colitis (UC) or familial adenomatous polyposis between 2004 and 2018. Data were collected from a prospectively, surgeon-maintained database. Pouch function and quality of life scores were obtained via validated questionnaires. A multivariate analysis was utilised to explore predictors of quality of life and pouch function.

**Results:**

The study reports 105 patients who underwent IPAA with a covering ileostomy. The majority of operations were performed for UC (97, 92.4%). The median age of patients was 36 years and the male to female ratio was 1:1. Thirty patients (28.5%) suffered early post-IPAA complications, while pouch failure rate was 11.4% (12/105). Late complications were reported at a rate of 45%. On long-term follow-up, the median Pouch Function Score was 7 (IQR 3–14). Both the physical and mental sections of the quality of life score were at a median indistinguishable from the normal population but had different predictors associated with them.

**Conclusion:**

Our findings recognise the complex interplay between physical and psychological wellbeing after pouch surgery and advise psychological counselling where appropriate.

## Introduction

Restorative proctocolectomy with ileal pouch anal anastomosis (IPAA) has become a standard treatment option for patients with ulcerative colitis (UC) and familial adenomatous polyposis (FAP). Technical advances and refinement of technique have led to a decrease in morbidity and mortality associated with the surgery.^1^ While this procedure offers the benefit of avoiding a permanent ileostomy, it also has specific complications and long-term sequelae.^1–3^ While previous studies have shown good long-term quality of life outcomes following IPAA,^3–5^ it is important to continue analysing the physical and psychological wellbeing and long-term complications associated with this operation.

In this study, we present our experience over a period of 14 years, adding to the existing body of evidence on this topic. The aim of this paper is to examine the long-term quality of life outcomes following IPAA procedures, specifically analysing the relationship between physical and mental wellbeing. Additionally, we will examine various factors that influence the quality of life following surgery.

## Methods

### Study design

This study is a single-centre retrospective study that was conducted in a specialised colorectal surgery unit at a tertiary care university hospital in the United Kingdom. The study adhered to the ethical principles of medical research outlined in the Helsinki Declaration and was reported in accordance with the STrengthening the Reporting of Observational studies in Epidemiology (STROBE) guidelines.^6,7^ The study received approval from the local audit department.

### Patient selection

Patients who underwent a two- or three-staged panproctocolectomy with a defunctioning ileostomy for ulcerative colitis, indeterminate colitis or FAP between the years 2004 and 2018 were included in the study. Those who had a one-stage operation (one patient, so not suitable for analysis) and those with a significant lack of clinical records were excluded.

### Standards of care

As per department protocol, each patient underwent a careful evaluation by dedicated inflammatory bowel disease (IBD) surgeons, gastroenterologists and pathologists before the creation of the pouch. In the case of FAP, a multidisciplinary approach was employed to evaluate and decide on the need to perform IPAA. The operations were performed either laparoscopically or via open technique, depending on the surgical expertise. A two-staged procedure involved a panproctolectomy in the initial setting, together with an IPAA and a defunctioning ileostomy that was subsequently reversed. A three-staged procedure comprised an initial subtotal colectomy followed by a completion proctectomy, IPAA and a defunctioning ileostomy, which again was reversed when deemed appropriate. A J-pouch was fashioned in all cases and IPAA was performed using either a hand-sewn or stapled technique, depending on the surgeon's preference or clinical circumstance. A defunctioning loop ileostomy was performed in all patients. Prior to the closure of the ileostomy, a contrast pouch enema study and examination under anaesthesia was performed to assess the integrity of the IPAA. Flexible pouchoscopy was performed in selected cases prior to the closure of the ileostomy. The pouch operations were all performed by dedicated IBD surgeons. All patients were followed up by IBD nurses, gastroenterologists and the colorectal surgeon in clinic, routinely, according to hospital protocol.

### Data collection

Data were collected retrospectively from a surgeon-maintained confidential database and medical records. Both paper and electronic records were assessed by at least two members of our team. Demographic information, operative details, complications, follow-up data, pouch function/failure and quality of life scores were collected for each patient. Complications were divided into pouch-related and stoma-related. Pouch-related complications were further divided into early (leak/collection) and late (pouchitis, cuffitis and need for biologics/antibiotic therapy) complications. Anastomotic leaks and collections were diagnosed and defined based on clinical, radiological and/or surgical assessments. Ileostomy-related small bowel obstruction and hernias were diagnosed using clinical parameters and cross-sectional imaging as appropriate. High-output ileostomy was defined as those with an output of greater than 1 litre in a 24-hour period, ileostomy output that resulted in electrolytic abnormalities and those that resulted in delayed discharge from the hospital. Pouch failure was defined as the failure to reverse a defunctioning ileostomy, subsequent defunctioning of a failing pouch, or pouch excision and a permanent ileostomy (as per the Association of Coloproctology of Great Britain and Ireland (ACPGBI)).^8^ Pouch function was assessed using the pouch function score (PFS) developed by Lovegrove *et al.*^9^ The 12-item Short Form Survey (SF-12) was used to score quality of life.^10^ Both the PFS and the SF-12 scores were collected via telephone-mediated questionnaires at a single time point in early 2021 as part of standard follow-up. The two components of the score (Mental Component Score (MCS-12) and Physical Component Score (PCS-12)) were analysed separately.

### Outcomes measured

The primary outcome was to explore predictors of quality of life and pouch function. For this, a univariate analysis of different variables against the two components of the SF-12 was carried out. Variables that showed statistical significance were then included in a subsequent multivariate analysis.

### Statistical analysis

Categorical variables were presented as counts and percentages/ratios. Continuous variables were presented as medians and interquartile ranges (IQR). For the univariate analysis, Pearson's Chi-Square test or Fisher's exact test were used to compare categorical variables while Spearman's correlation or Wilcoxon rank-sum test were used to compare continuous variables. Multiple linear regression analysis was used for the multivariate analysis. A *p*-value of less than or equal to 0.05 was considered statistically significant. Statistical analysis and graphic presentations were performed using R software (version 4.0.4, R Foundation for Statistical Computing, Vienna, Austria).

## Results

A total of 132 patients underwent IPAA with a defunctioning ileostomy. However, 26 patients were excluded owing to a lack of clinical records, and one patient was excluded from the study as they had a single-stage operation. A total of 105 patients were followed up with a median follow-up of 8 years (IQR 6–12).

### Baseline demographics

Of the 105 patients studied, there were 45 two-stage and 60 three-stage procedures. The majority of operations were performed for colitis (97, 92.4%) and the remaining for FAP. Some 69.5% (73/105) of the operations were performed using an open technique – especially during the early period of the study. The baseline demographics of patients in the cohort are depicted in Table 1.

**Table 1:** Basic demographics, and operative and complications details

**Basic demographics**	
Median age, years (IQR)	36 (29–47)
Sex (male:female)	1:1
Primary pathology (UC, FAP)	97 (92.4%), 8 (7.6%)
Approach (laparoscopic, open)	32 (30.5%), 73 (69.5%)
Operation (two-, three-staged)	45 (42.9%), 60 (57.1%)
**Outcomes**	
Pouch failure	12 (11.4%)
Time to stoma closure, months (IQR)	5, (4–7)
Early pouch complications (leak/collection)	30 (28.6%)
Stoma complications	60 (57.1%)
**Late pouch complications**	
Pouchitis	45 (42.9%)
Cuffitis	9 (8.6%)
Use of biologics	3 (2.9%)
Use of antibiotics	41 (39%)
**Functional/quality of life scores**	
Median pouch function score (IQR)	7 (3–14)
PCS12 (IQR)	51 (42–57)
MCS12 (IQR)	52 (43–59)
Follow-up time, months (IQR)	96, (72–144)

FAP: familial adenomatous polyposis; UC: ulcerative colitis

### Outcomes

Thirty patients (28.5%) suffered early post-IPAA complications. The leak rate following an IPAA was 11.4% (12/105). Ten of these patients were managed conservatively, one with computed tomography-guided drainage, and one patient underwent a re-operation in the form of a laparoscopic washout, pouch excision and formation of end ileostomy.

Forty-seven patients (44.8%) developed pouchitis (45, 42.9%) and/or cuffitis (9, 8.6%) during the follow-up period. Most of these patients (41, 87%) required antibiotic therapy to manage their symptoms, and only three patients needed biologic therapy (Table 1).

The rate of ileostomy-related complications was 42% (44 cases); the most common being dehydration (25%) and small bowel obstruction at the stoma site (26.8%). Four (3.8%) of ileostomy closures were complicated by an anastomotic leak from the closure itself and all of these warranted surgical re-explorations. Out of the 105 patients, 98 (93.3%) had their ileostomy closed. The median time to closure of stoma was 5 months (IQR 4–7). Four patients did not have their ileostomy reversed for various reasons including patient choice, death due to other causes and pelvic sepsis.

On long-term follow-up, our pouch failure rate was 12/105 (11.4%). Eight patients had their pouch excised over the study period due to chronic pelvic sepsis, pouch strictures, chronic fistulae and anastomotic leaks. We collected PFS and SF-12 score for 72 patients (69%) during the follow-up period. The median PFS was 7 (IQR 3–14). Median PCS-12 was 51 (42–57) and median MCS-12 was 52 (IQR 43–59) (Table 1).

### Primary outcome analysis

On univariate analysis, four variables were found to be significantly associated with worse PCS-12. These were: stoma complications (*p*=0.02), pouchitis (*p*=0.003), pouch failure (*p*<0.001) and higher PFS (*p*<0.001). For MCS-12, we detected seven variables that impacted the score negatively. These were: female sex (*p*=0.005), younger age (*p*=0.006), laparoscopic approach (*p*=0.008), early pouch complications (*p*=0.009), pouch failure (*p*=0.0007), shorter follow-up time (*p*=0.008) and higher PFS (*p*=0.02) (Table 2). Upon multivariate analysis, stoma complications, pouchitis and pouch failure significantly affected a patient’s physical quality of life (as measured using the PCS-12 questionnaire). The overall model *p*-value was less than 0.001 with adjusted *R*-squared of 0.35. Female gender, young age, pouch failure, high PFS and early pouch complications affected the patient’s mental wellbeing (as measured using the MCS-12 questionnaire) (model *p*-value <0.001, adjusted *R*-squared=0.52). Analysis of the predictors of PFS showed significant association with late pouch complications (pouchitis and antibiotics use) (Table 2).

**Table 2:** Univariate analysis of predictors of quality of life and pouch function

	PCS-12 [median (IQR), *p*-value]	MCS-12 [median (IQR), *p*-value]	PFS [median (IQR), *p*-value]
Sex M:F	53 (47–57):49 (40–56), 0.33	58 (49–60):48 (35–56), **0.005**	7 (2–14):6 (3–14), 0.83
Age (*p*-value)	0.4	**0.006**	0.11
UC vs FAP	51 (42–57):47 (39–57), 0.6	53 (44–59):46 (24–58), 0.5	7 (2–14):8 (5–27), 0.25
Two vs three-stage	49 (45–56):52 (41–57), 0.5	50 (43–57):55 (44–59), 0.5	8 (4–14):5 (2–12), 0.15
Lap vs open	47 (41–56):51 (43–57), 0.4	45 (29–56):55 (46–60), **0.008**	7 (5–15):5 (2–13), 0.2
Pouch failure	37 (30–39):52 (46–57), **0.0009**	24 (20–30):54 (46–59), **0.0007**	28 (19–28):7 (2–11), **0.005**
Pouch complications	50 (40–56):52 (46–57), 0.7	45 (30–54):51 (47–60), **0.009**	7 (3–15):7 (3–13), 0.7
Stoma complications	49 (40–55):56 (48–57), **0.02**	52 (43–59):53 (43–59), 0.9	8 (3–15):5 (2–8), 0.2
Time from IPAA to questionnaire (*p*-value)	0.9	**0.008**	0.5
Pouchitis	48 (38–52):55 (48–57), **0.003**	52 (42–58):50 (43–59), 0.6	10 (6–16):3 (2–8), **0.0004**
Cuffitis	50 (44–52):51 (42–57), 0.5	43 (40–54):53 (46–59), 0.1	13 (7–15):7 (3–11), 0.1
Biologics	42 (39–45):51 (42–57), 0.2	53 (53–53):52 (43–59), 0.9	13 (10–16):7 (3–14), 0.3
Antibiotics	50 (39–54):54 (46–57), 0.06	53 (46–58):50 (42–59), 0.9	10 (7–16):4 (2–9), **0.002**
Pouch Function Score (*p*-value)	**0.00004**	**0.02**	—
Time to closure (*p*-value)	0.8	0.08	0.4

FAP: familial adenomatous polyposis; IPAA: ileal pouch anal anastomosis; MCS-12: Mental Component Score; PCS-12: Physical Component Score; PFS: pouch function score; UC: ulcerative colitis

## Discussion

The fundamental purpose for performing IPAA is to improve the patient's sense of wellbeing. The most common ways of assessing the same would involve studying the long-term pouch function and the quality of life.^11,12^ Quality of life assessment has two components, physical and mental wellbeing scores. The low morbidity associated with the procedure shifted the focus towards assessing the long-term quality of life outcomes. As is evident in this paper, only 1% of the patients who were followed up with a median follow-up of 8 years developed severe complications, and only one patient warranted emergency pouch excision for a leak. The overall pouch failure rate (as defined by the ACPGBI) was 11% over the long-term period, which is in keeping with the literature.^1,13^ In this single-centre study, all patients having a pouch had defunctioning temporary ileostomy. Interestingly, more complications were attributed to the ileostomy reversal rather than the pouch formation procedure. However, we cannot comment on pouch complications if the stoma had not been created.

The long-term results of this paper suggest that quality of life following restorative proctocolectomy and IPAA is indistinguishable from the normal population. The median for both components of our quality of life score were similar to the normal population (50, 51 and 52 for normal, PCS-12 and MCS-12, respectively). Our findings corroborate the findings of Heikens *et al*, who reported in a large study that patients’ perception of their health and wellbeing improves with time and reaches those of the normal population on long-term follow-up.^14^

Although several publications investigated predictors of quality of life after IPAA,^5,15–17^ our approach is unique in that it addressed the physical and mental aspects separately. This approach revealed some interesting new insights. The mental and physical health survey questionnaire are assessed separately using MCS-12 and PCS-12 scores. The physical quality of life is assessed by gauging whether a patient is able to perform certain activities, has pain or needs to avoid certain kinds of work. On the other hand, mental quality of life focusses on the patient's contentment with life, feeling energetic and being at peace with themselves without having any social inhibitions.^10^

**Figure 1:**
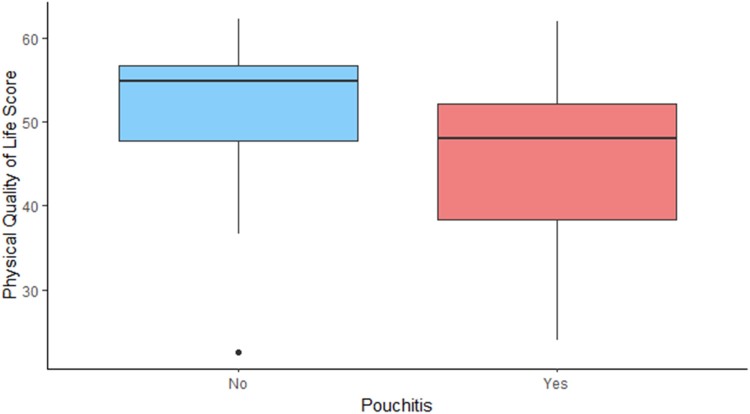
Impact of pouchitis on physical quality of life

**Figure 2:**
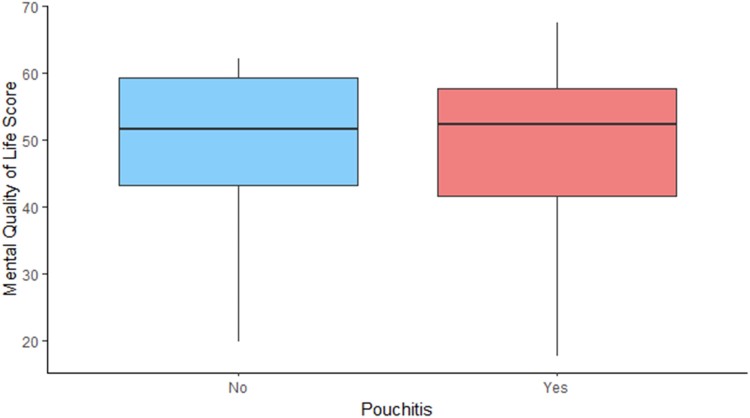
Impact of pouchitis on mental quality of life

**Figure 3:**
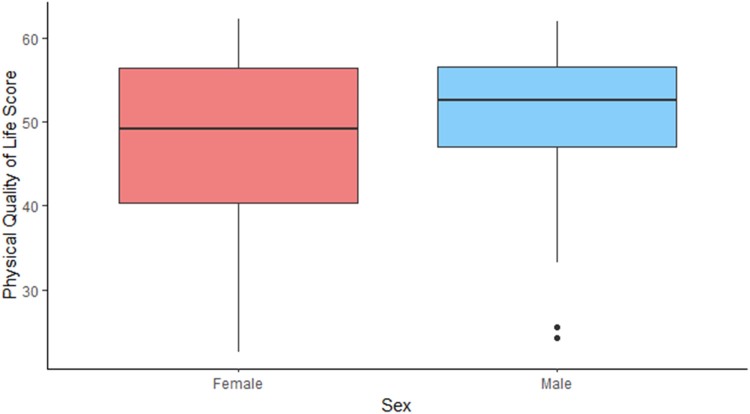
Physical quality of life by patient's sex

**Figure 4:**
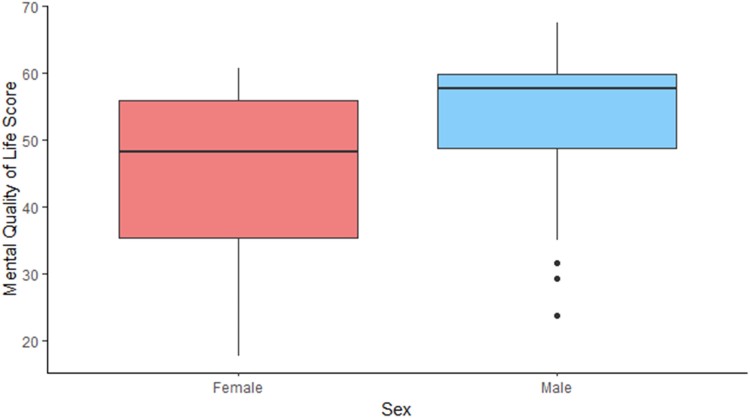
Mental quality of life by patient's sex

It is not surprising that poor pouch function and pouch failure are associated with worse quality of life. However, there seems to be a dissociation between the physical and mental component of the score on a number of variables. An example of this is the impact of pouchitis and stoma complications. Our analysis shows that they affect the physical status of the patients – in concordance with previous studies.^11,18^ However, it does not seem to impact mental wellbeing significantly, which could suggest that it is well tolerated psychologically (Figures 1 and 2). In contrast, young age, female sex and early pouch complications had the opposite effect (Figures 3 and 4).

The univariate analysis has revealed some interesting trends that lost significance on the subsequent multivariate analysis. Of these, the worst MCS-12 is in laparoscopic cases. Intuitively, surgeons associate the laparoscopic approach with a better cosmetic outcome and better body image for the patient.^19^ This is not the trend in our analysis. On further analysis, we could explain this by the shorter follow-up in laparoscopic cases. The other interesting finding in the univariate analysis was the impact of follow-up on MCS-12. Our results suggest better mental wellbeing with time since surgery, implying that patients mentally come to peace with their pouches over many years rather than immediately after the procedure. There is paucity in publications exploring the psychological impact of surgery. The impact of patient demographics on mental health is not well understood. O’Hara *et al* presented evidence that young, male patients from a low social background are at a higher risk of postoperative mental distress.^20^ Social background data were not available for our analysis, but our results show a worse mental wellbeing score despite similar physical wellbeing score in young females. It is conceivable that young patients would show less resilience against a life-changing surgery. However, the impact of the patient's sex is not fully understood.

**Figure 5:**
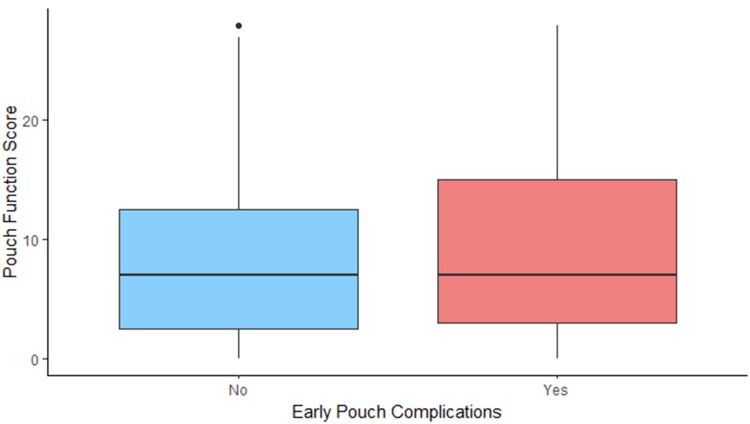
Impact of early pouch complications on pouch function

**Figure 6:**
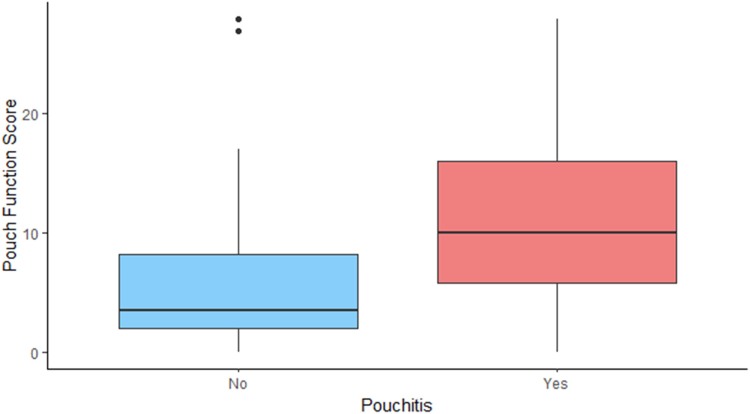
Impact of pouchitis on pouch function

The analysis of the PFS revealed a couple of interesting points. Early pouch complications were not associated with long-term worse function, while late pouch complications (pouchitis, cuffitis, antibiotics use) significantly impacted pouch function (Figures 5 and 6). This indicates that a well-managed early pouch leak/collection may not have a long-lasting impact on the pouch function and longevity. Also, the duration of having the pouch did not affect long-term function.

There are several limitations to this study. Its retrospective nature introduces the known biases related to data accuracy. Despite our best efforts, critical data were missing on many of the cases, leading to a relatively high exclusion rate. We also did not have preoperative base line scores, but this is unlikely to be relevant given that the median PCS-12 and MCS-12 were very close to the normal population. The functional and quality of life assessment was performed at a single time point, as it is well understood that pouch function and a patient's assessment of wellbeing changes with time. This is a confounder that has to be factored in.

Despite these limitations, our study provides new insights into the psychological wellbeing of patients having pouch surgery. The findings of this paper will enable clinicians to better recognise the complex interplay between physical and psychological wellbeing postoperatively and advise psychological counselling where appropriate.

## Conclusion

The quality of life of patients following restorative proctocolectomy and IPAA is indistinguishable from the normal population in the long-term follow-up. The findings of this paper recognise the complex interplay between physical and psychological wellbeing after pouch surgery. There appears to be a dissociation between mental and physical wellbeing, with this paper identifying a subgroup of patients who might have worse mental quality of life and therefore benefit from focused support on a long-term basis after surgery.

## Ethics approval and consent to participate

Ethical review or individual patient consent was not required as non-interventional, retrospective data were used. The online National Research Ethics Service decision tool was used to confirm the same.

## References

[1] Ng K-S, Gonsalves SJ, Sagar PM. Ileal-anal pouches: A review of its history, indications, and complications. *World J Gastroenterol* 2019; **25**: 4320–4342 31496616 10.3748/wjg.v25.i31.4320 PMC6710180

[2] Baker DM, Folan A-M, Lee MJ *et al.* A systematic review and meta-analysis of outcomes after elective surgery for ulcerative colitis. *Colorectal Dis* 2021; **23**: 18–33. 32777171 10.1111/codi.15301

[3] Fazio VW, Kiran RP, Remzi FH *et al.* Ileal pouch anal anastomosis: analysis of outcome and quality of life in 3707 patients. *Ann Surg* 2013; **257**: 679–685. 23299522 10.1097/SLA.0b013e31827d99a2

[4] Cherem-Alves A, Lacerda-Filho A, Alves PF *et al.* Surgical results and quality of life of patients submitted to restorative proctocolectomy and ileal pouch-anal anastomosis. *Rev Col Bras Cir* 2021; **48**: e20202791. 33787765 10.1590/0100-6991e-20202791 PMC10683452

[5] Carcamo L, Miranda P, Zúñiga A *et al.* Ileal pouch-anal anastomosis in ulcerative colitis: outcomes, functional results, and quality of life in patients with more than 10-year follow-up. *Int J Colorectal Dis* 2020; **35**: 747–753. 32067061 10.1007/s00384-020-03529-7

[6] World Medical Association. World medical association declaration of Helsinki: ethical principles for medical research involving human subjects. *JAMA* 2013; **310**: 2191–2194. 24141714 10.1001/jama.2013.281053

[7] von Elm E, Altman DG, Egger M *et al.* The strengthening the reporting of observational studies in epidemiology (STROBE) statement: guidelines for reporting observational studies. *Int J Surg* 2014; **12**: 1495–1499. 25046131 10.1016/j.ijsu.2014.07.013

[8] ACPGBI. ACPGBI Ileoanal Pouch Report. 2017.

[9] Lovegrove RE, Fazio VW, Remzi FH *et al.* Development of a pouch functional score following restorative proctocolectomy. *Br J Surg* 2010; **97**: 945–951. 20474005 10.1002/bjs.7021

[10] Michalos AC. *Encyclopedia of Quality of Life and Wellbeing Research*. Dordrecht: Springer Netherlands; 2014.

[11] Ardalan ZS, Sparrow MP. A Personalized Approach to Managing Patients With an Ileal Pouch-Anal Anastomosis. *Front Med* 2019; **6**: 337. 10.3389/fmed.2019.00337 PMC7000529 32064264

[12] Murphy PB, Khot Z, Vogt KN *et al.* Quality of life after total proctocolectomy With ileostomy or IPAA: A systematic review. *Dis Colon Rectum* 2015; **58**:899–908. 26252853 10.1097/DCR.0000000000000418

[13] Leinicke JA. Ileal pouch complications. *Surg Clin North Am* 2019; **99**: 1185–1196. 31676057 10.1016/j.suc.2019.08.009

[14] Heikens JT, de Vries J, van Laarhoven CJHM. Quality of life, health-related quality of life and health status in patients having restorative proctocolectomy with ileal pouch-anal anastomosis for ulcerative colitis: a systematic review. *Colorectal Dis* 2012; **14**: 536–544. 21176062 10.1111/j.1463-1318.2010.02538.x

[15] Watanabe K, Nagao M, Suzuki H *et al.* The functional outcome and factors influencing the quality of life after ileal pouch anal anastomosis in patients with ulcerative colitis. *Surg Today* 2018; **48**: 455–461. 29234962 10.1007/s00595-017-1613-8

[16] Lavryk OA, Stocchi L, Hull TL *et al.* Factors associated with long-term quality of life after restorative proctocolectomy with ileal pouch anal anastomosis. *J Gastrointest Surg* 2019; **23**: 571–579. 30097964 10.1007/s11605-018-3904-9

[17] Lee GC, Deery SE, Kunitake H *et al.* Comparable perioperative outcomes, long-term outcomes, and quality of life in a retrospective analysis of ulcerative colitis patients following 2-stage versus 3-stage proctocolectomy with ileal pouch-anal anastomosis. *Int J Colorectal Dis* 2019; **34**: 491–499. 30610435 10.1007/s00384-018-03221-x PMC6450759

[18] Koerdt S, Jehle EC, Kreis ME, Kasparek MS. Quality of life after proctocolectomy and ileal pouch-anal anastomosis in patients with ulcerative colitis. *Int J Colorectal Dis* 2014; **29**: 545–554. 24370856 10.1007/s00384-013-1814-6

[19] Larson DW, Davies MM, Dozois EJ *et al.* Sexual function, body image, and quality of life after laparoscopic and open ileal pouch-anal anastomosis. *Dis Colon Rectum* 2008; **51**: 392–396. 18213489 10.1007/s10350-007-9180-5

[20] O’Hara MW, Ghoneim MM, Hinrichs JV *et al.* Psychological consequences of surgery. *Psychosom Med* 1989; **51**: 356–370. 2734428 10.1097/00006842-198905000-00010

